# Defining mangrove-fisheries: A typology from the Perancak Estuary, Bali, Indonesia

**DOI:** 10.1371/journal.pone.0249173

**Published:** 2021-04-21

**Authors:** Rachel Seary, Tom Spencer, Mike Bithell, Chris McOwen, Yoshitaka Ota

**Affiliations:** 1 Cambridge Coastal Research Unit, Department of Geography, University of Cambridge, Cambridge, United Kingdom; 2 UN Environment Programme World Conservation Monitoring Centre (UNEP-WCMC), Cambridge, United Kingdom; 3 School of Marine & Environmental Affairs, College of The Environment, University of Washington, Seattle, WA, United States of America; Tanzania Fisheries Research Institute, UNITED REPUBLIC OF TANZANIA

## Abstract

This study develops a definition of what mangrove-fisheries can encompass, incorporating a broad range of their possible characteristics. A detailed case study was conducted to develop a typology of mangrove-fishing in the Perancak Estuary, Bali, Indonesia, using interview surveys to investigate the fishing activities associated with mangroves. This case study demonstrated the complexity that a mangrove-fishery can entail, where fishing is connected to the mangrove forest by fishers of multiple sectors, functions, locations and temporal scales. Through a comparison with other mangrove-fishing communities in Bali, it also highlighted that mangrove-fisheries are variable even when in close proximity. With particular reference to this case study, a framework was developed as a flexible tool for identifying the multiple dimensions of a mangrove-fishery in a local context. Following this framework should encourage researchers and managers to look outside of the groups of fishers traditionally expected to benefit from mangrove fishing. This will enable the development of a broader definition of mangrove-fisheries in a site specific way. Identifying the full scope of fishers that contribute to or benefit from a mangrove-fishery is the first step towards building management measures that reflect the interests of groups of fishers that may otherwise remain under-represented. This is in line with international efforts for sustainability, especially in promoting small-scale fishers’ access to sustainable resources under the UN Sustainable Development Goals.

## 1 Introduction

Efforts in mangrove management to date have focussed upon slowing mangrove forest loss, restoration and climate mitigation, to the detriment of a consideration of the social dimensions of mangrove use for fisheries. In part this stems from the lack of ubiquitous definition of what constitutes a mangrove-fishery; studies so far have used broad and sometimes vague descriptions of mangrove-fishing. On one hand, research has described traditional fishers collecting directly from the mangrove [1–3]. On the other hand, other studies have solely linked offshore catch to mangrove presence inshore. Furthermore, fisheries data in areas where mangroves occur is typically poor, making it difficult to quantify use. Where they coexist with larger scale fisheries, small-scale fisheries in general are often under-represented in data collection, fishery reports and management plans [4–6].

Mangrove-associated fishing, whether it be directly within the mangrove or deriving indirect benefits, can contribute greatly to the livelihoods of coastal communities. However with limited quantitative data on the nature of the mangrove–fishery association, it is difficult to be sure how the economic or societal importance of mangrove-associated fisheries compares to larger industrial fisheries (which are more prevalently reported and represented than small scale activities). This is further complicated by the fact that what constitutes a mangrove-fishery may vary in space and time, making broad scale generalisations difficult and somewhat uninformative. To address these questions, this study develops a typology of what a mangrove-fishery *can* encompass. This is important not only in revealing the hidden monetary or societal value of mangroves for fishers and but also in fully understanding “nature’s contribution to people” (as conceptualized by IPBES [7]) and supporting the adaptive and inclusive management of the marine environment. Information on the spatial and temporal characteristics of mangrove-fishing livelihoods, including aspects outside of the mangrove habitat, will also contribute to the knowledge needed to successfully implement marine spatial planning and other spatial management measures. This information will therefore be relevant to the ongoing efforts by governments internationally in meeting the UN Sustainable Development Goals (SDGs), particularly those SDGs concerning sustainable use of the seas (SDG 14) as well as livelihood centred SDGs, such as ending poverty (SDG 1) and hunger (SDG 2) [7].

### 1.1 How are mangrove-fisheries currently characterised?

Fisheries associated with mangroves, particularly within the quantitative literature, are rarely described using the term “mangrove-fisheries”. Fisheries are most often named by their target species, sector or location (Table 1), for example the “commercial shrimp fishery” [8–10] or the “Gulf fishery” [11]. From publications where the value of mangroves to fisheries has been quantified, Table 1 documents the range of characteristics that have been used to describe fisheries associated with mangroves. As demonstrated in Table 1, quantifications of mangrove-fishery value until now have been based upon a wide range of scope regarding i) where mangrove fishing takes place, ii) which catch is included and iii) who is fishing.

**Table 1:** Characteristics used to describe mangrove-associated fishing in the quantitative literature by fishing location, species included from catches (mangrove associated or otherwise), fishing sectors included, fishing gear used and how the fishery is identified. The list of papers explored, representing studies which have quantified mangrove-fishery linkages, were compiled by Carrasquilla-Henao et al. [12].

Authors	Fishing location	Species included	Sector	Fishing gears used	Study location	Identity of fishery
[13, 14]	Half degree sections of the coast (30 nautical miles)	Mangrove Estuarine Other	Commercial	Trawl, line, net, pot	East Coast of Australia	By gear
[15]	0–4026.81 m from mangrove	5 selected species of mangrove associates	Artisanal	Hooks, rings, spear, cast net, gill net, clam digging	San Igancioe Navachiste-Macapule Lagoon system, Mexico	By gear By sector
[16]	Estuary Offshore Coastal	Estuary	Commercial	Trawl, net, pot, line	Coast of Queensland, Australia	By gear
[17]	Coastal	Mangrove	Commercial	Not specified in paper	Peninsula Malaysia	By sector
[18]	Sites including coral reefs and seagrass	Mangrove	Artisanal Commercial	Not specified in paper	Coastal provinces of the Philippines	By sector
[19]	Estuary	All except exclusively oceanic species	Commercial	Not specified in paper	New South Wales, Australia	Not specified in paper
[20]	Estuary	All	Commercial Recreational	NA (fisheries independent survey)	East Australia	Not specified in paper
[21]	Inshore Offshore	Mangrove	Commercial	Trawler	27 regional locations worldwide	Not specified in paper
[10]	Within mangrove Adjacent offshore areas	Penaeid shrimp	Commercial	Within mangrove: tidal traps Offshore: Not specified	Indonesia by province	By target By sector
[11]	Mud-mangrove banks	5 penaeid prawn species	Commercial	NA (fisheries independent survey)	Embley River, NE Gulf of Carpentaria, Australia	By region
[9]	Not specified	All penaeid shrimp: Mangrove Other	Commercial Artisanal	Not specified in paper	37 countries worldwide	By target
[8]	Offshore Coastal Mangrove inlets	Penaeid prawns	Commercial	Not specified in paper	Peninsula Malaysia	By target
[22]	Nearshore	Marine prawns	Not specified in paper	Not specified in paper	37 countries worldwide	Not specified in paper
[23]	Coastal Estuary	All prawn species: Mangrove Other	Commercial	Trawler, bag net, paired trawl, drift/gill net, trammel net	Western Peninsula of Malaysia,	By target By location
[24]	Offshore	Shrimp only: Mangrove	Commercial Artisanal	Not specified in paper	Campeche State, Mexico	By location By target
[25]	Coastal	Mangrove	Artisanal	Not specified in paper	The West Coast of Malaysia mangrove area	By target By sector
[26]	Estuarine survey (Fishery occurs: Offshore Estuary Mangrove)	2 prawn species: Estuary	Commercial	NA (fisheries independent survey)	Joseph Bonaparte Gulf, NW Australia	By fishing location
[27]	Within mangrove sampling regarding offshore fishery	Single prawn species: Mangrove	Commercial	NA (fisheries independent survey)	Mangrove estuaries, NE coast of Australia	By fishing location By target
[28]	Nearshore Offshore	All species caught	All species caught	Not specified in paper	Southern Region of Vietnam	By fishing location
[29]	Within 50 km of mangrove incl. Mangrove Offshore reefs Sandy bottoms	Mangrove	Small-scale	Hand line, gill net	Gulf of Mexico	By sector By practice
[30]	Coastal shelf area bordering mangroves	Species observed in the mangrove	Subsistence Commercial	Commercial: Beach seine, muro-ami, gill net, otter trawl Subsistence: Not specified in paper	Philippines	By sector
[31]	Riverine	6 species (not specified in paper)	Commercial Recreational	Not specified in paper	Alvarado Lagoon System, Gulf of Mexico	By location By sector By cooperative

* One paper included in the list by [12] was not accessible; [32].

In terms of location, assessments have often been made based on arbitrary effect distances to mangrove or related to the boundaries of known fishing locations, rather than in relation to any ecological parameters of the mangrove habitat itself. In terms of catch, available fisheries landings data can also lack species specific information, meaning the proportion of true mangrove-associate species catches is unknown. Much of the literature surrounding mangrove-fisheries, particularly the grey literature, has stated that 75% of fisheries catches are mangrove-associated, without any ecological evidence for this statement [33]. This assumption has been criticized on two fronts: i) that the proportion of species that use mangroves is much less than this figure, and ii) that the statement is too generalised for what is a site-specific relationship [33].

In terms of who is fishing, quantification of mangrove-value is often limited to the fishing sectors for which data is available. At best, a maximum of two fishing sectors involved in mangrove-fishing have been studied in a single location (Table 1). Small-scale fishing, such as artisanal and subsistence fishing, has been better addressed in the qualitative literature [34–36], with quantitative measurements of mangrove-associated fishing predominantly relating to the commercial fishing sector (Table 1). This division means that the contribution of small-scale fisheries is not represented in quantitative measures of mangrove-fishery value. As a wide range of fishing sectors are reported to use mangroves across these studies (Table 1), it is possible that additional groups of mangrove users exist in a location where just one or two sectors have been included analytically. Thus, the development of a framework which details the range of characteristics a mangrove-fishery can encompass could contribute towards a more consistent, yet holistic, description and thus a more complete quantification of their benefits.

### 1.2 Aims and objectives

The aim of this study is to build a comprehensive typology of mangrove-fisheries; addressing some of the shortcomings relating to the existing characterisation of mangrove-fisheries described above. The development of characteristics which describe mangrove-fisheries thus makes efforts to include stakeholders in a mangrove-fishery outside of those sectors that are specifically identified/named as mangrove-fishers or mangrove-fisheries. In doing so it identifies and represents a broader range of groups interacting with, or benefiting from, mangroves than the limited sectors studied thus far. The recording of mangrove-associated fishing locations adds key information in forming this typology. Furthermore, only catches listed as known mangrove-associates in FishBase [37] or SeaLifeBase [38] are included within the development of a definition of mangrove-fishing.

The typology is based on the Perancak Estuary, Jembrana sub-district, Bali, Indonesia. Indonesia has the largest area of mangrove of all countries worldwide, however it has also experienced the largest area loss [39]. Bali, and specifically the Perancak Estuary, has been the focus of research reporting rapid mangrove loss due to aquaculture conversion [40–43]. The region is now experiencing a period of greening (increasing mangrove area) due to natural regrowth and replanting efforts [42]. This location is representative of the pattern of aquaculture establishment and subsequent land cover change which occurred from the 1970s to the 1990s in many areas of Indonesia (Gusmawati et al. 2018, Proisy et al. 2018), wider Asia and worldwide [44]. Despite this changing mangrove extent, the importance of the mangrove-fishing activities that exist in Perancak Estuary area have yet to be studied and no data on mangrove-fishing prior to mangrove loss exists. While our study concentrates on the characteristics mangrove-fisheries exhibits in Bali, we use our findings to develop a universal framework which can be used flexibly to inform and be modified to the characteristics of mangrove fishing in the many other regions that mangroves occur.

## 2 Methods

The research involved human participants (interviews with fishers) and therefore an ethics assessment was conducted which was reviewed and approved by the Ethics Review Chair at the Department of Geography, University of Cambridge. The review number was 340 and it was approved on 16/01/2017. Consent of participants was obtained orally.

### 2.1 Site description

#### 2.1.1. Geological and ecological setting

The Perancak Estuary covers an area of 7.55 km^2^ [40]. The estuary has 4 main branches, fed by mountain catchments at its northern boundary and terminating at its southern limit in the Indian Ocean. The estuary exhibits a semi-diurnal tide, with an estimated tidal range of 2m [45]. The land surrounding the estuary can be submerged by 0.5-1m of estuarine water at high tide, particularly on spring tides [45]. The region experiences a dry season between April and October and a rainy season from November to March, with an annual average rainfall total of 1500 mm. The monthly average temperature in Bali ranges from 29–32°C [46].

In a 2015 estimate, the Perancak Estuary was surrounded by 1.25 km^2^ (125 ha) of mangrove [40]. This estimate followed a relatively recent increase in mangrove extent, in response to cutting throughout the 2001–2003 period which left just 0.4 km^2^ of mangrove. Of the 1.25 km^2^ of mangrove area, approximately 0.35 km^2^ is replanted mangrove on aquaculture pond walls and floors while the remainder was naturally colonized or re-colonized [40]. Mangrove species composition varies considerably between natural and replanted areas; in natural forest plots in 2015, Proisy et al. (2017) found that *Avicennia alba* (70% of plots) was the most commonly occurring species, followed by *Sonneratia alba* (50%) and *Avicennia officinalis* (37.5%). By contrast, planted plots were dominated by *Rhizophora* spp. (*Rhizophora apiculata* (53%), *Rhizophora mucronata* (40%) and *Rhizophora stylosa* (33%) which were not found in natural plots [40].

There are 1,546 aquaculture ponds surrounding the Perancak Estuary, covering 3.6 km^2^. Ponds in the area include fish culture, intensive, semi-intensive and polyculture ponds (see Gusmawati et al. (2018) for pond locations and specifications [41]). Only 369 ponds are currently (2018) active and 70% have been abandoned [41]. Pond abandonment, following periods of low production, has been associated with the spread of disease through closely linked and densely populated ponds in the central Perancak Estuary area and specifically to the incidence of white spot disease, a viral infection that causes mortality of shrimp. The virus has been prevalent in Indonesia since its introduction to Java in 1994 and can be transmitted through water-sharing between ponds.

#### 2.1.2. Socio-economic context and fishing activities

**Fig 1:**
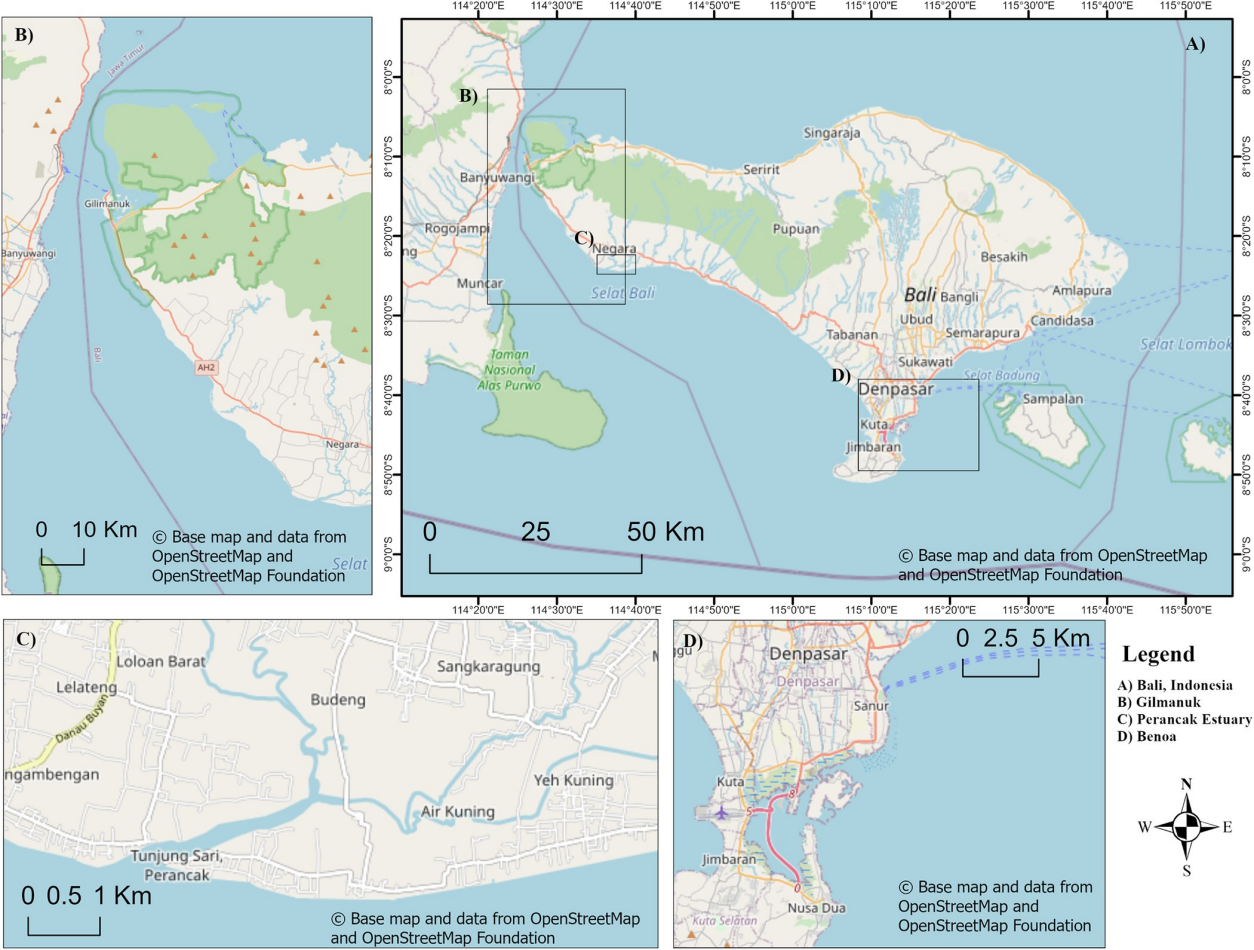
Location of case study sites showing A) Gilimanuk, West Bali B) Bali, Indonesia, C) The Perancak Estuary, Jembrana sub-district, Jembrana Regency, West Bali which was the primary study site and D) Denpasar City, South Bali, the location of the Benoa Fishing Village. The base map and data were sourced from OpenStreetMap and OpenStreetMap Foundation. This figure contains information from OpenStreetMap and OpenStreetMap Foundation, which is made available under the Open Database License. This map was created using ArcGIS Pro software.

The central Perancak Estuary lies within the Jembrana sub-district of the Jembrana Regency, which has 62,790 residents. Four main villages—Perancak, Air Kuning, Yeh Kuning and Budeng—neighbour the estuary (Fig 1A and 1C). Agriculture, particularly rice farming, and fisheries are the most prominent economic sectors in the Jembrana Regency [47]. The Regency has a 604 km^2^ marine area and is the largest producer of marine fish in Bali. Average fish consumption in 2014 across the Regency was 29 kg/capita/year [48]. According to the Jembrana Regency Government Fisheries and Forestry Services, the potential marine fish production is 57.9 tonnes annually, split between pelagic (93%) and demersal (7%) catch [48]. Most commercial fishing at sea uses purse seines or lift nets while small-scale fishing uses gill nets or hook and line in Jukung (traditional boats)/boats without motors (<5 GT weight). Within the Jembrana sub-district of the Regency, it is reported that there are 1,532 fishermen, of whom 80% list fishing as their primary occupation. Regional reports suggest there are 627 active fishing boats, consisting of 611 with outboard motors, 9 motor boats and 7 Jukung [48]. These statistics do not include traditional fishing occurring in the region and underestimate the number of small-scale fishing boats (Jukung). Moreover, no mention of mangrove-fishing or fishing existing in the mangrove-lined Perancak Estuary is made within these reports, despite anecdotal evidence to the contrary.

#### 2.1.3. Comparison with wider Bali

To compare the Perancak Estuary typology with mangrove-fishing activities across wider Bali, shorter visits were made to Benoa, South Bali and Gilimanuk, West Bali where mangroves are also present.

The fishing community in Benoa Village, Denpasar, South Bali (Fig 1C) comprises 96 fishermen and their families. Benoa has a 13.73 km^2^ expanse of fringing mangrove forest which runs along Benoa Bay, a 25–30 km^2^ tidally influenced lagoon [49, 50]. The community, which was officially established in 2009, is centred around a community-owned and managed restaurant. The enterprise buys products (crab and fish) collected from, or cultured in, the mangrove by the community and sells them within the tourist-targeted restaurant built on a boardwalk within the mangrove forest.

The Gilimanuk fishing community lies just outside the bounds of the Bali Barat National Park, Southwest Bali (Fig 1D). The park supports 3.1 km^2^ of fringing mangrove forest [51]. Unlike the two other study sites, the coastal zone of Bali Barat National Park also includes 0.4 km^2^ of seagrass habitat and 8 km^2^ of coral reef [46, 51]. The fishing community in Bali Barat has changed in recent years from a community conducting fishing as a primary occupation to one centred on tourism. 30 of 78 members of the fishing community still actively fish but the majority of fishermen now use their boats as tourist rentals.

### 2.2 Data collection and analysis

Semi-structured interviews with fishers were conducted between February-March 2017, during the less busy rainy season. Interviews were conducted with any individual involved in fish production who thus might derive benefit from the presence of mangroves. Respondents included fishers who self-identified from five sectors: traditional; recreational; small-scale; and commercial fishing, as well as aquaculture. Initial respondents were identified through researchers at the Bali Institute for Marine Observation (BPOL) and thereafter identified through snowballing. Interviews were mostly pre-planned, taking place at the home of the respondents but some opportunistic interviews with recreational and traditional fishers were conducted at fishing sites. Interviews lasted between 30 to 90 minutes.

Thirty-two interviews were conducted in total. In the Jembrana sub-district, 8 semi-structured interviews were conducted with traditional fishers, 6 with recreational fishers, 3 with small-scale fishers, 2 with “fish masters” (managers) of commercial fishing boats and 4 with aquaculture workers or owners. Less structured interviews were also conducted with members of the community thought to be able to offer a summary of fishing activities in the local mangrove area. Two interviews were conducted in this manner in the Jembrana sub-district, with a fishing agent (an agent managing 50 small-scale fishing boats) and a government official from the local government fisheries office.

Interviews in the Benoa sub-district involved 1 semi-structured interview and 4 less structured interviews. Community representatives (less structured interviews) were more accessible for interview than fishermen (for semi-structured interviews) during the short visits to Benoa as these had to be planned in advance. The semi-structured interview took place during a pre-planned tourist mangrove fishing trip. An unstructured interview in Denpasar was conducted with the Director of the Mangrove Information Centre (MIC) in Benoa which led to further unstructured interviews with 3 representatives of the Forest Police, the head of a fishing community in Benoa Village and with the owner of the mangrove fishing tours company and worker within mangrove crab culture, along with the community secretary. In Gilimanuk, an unstructured discussion with 5 members of a fishing community took place, as well as a semi-structured interview with one small-scale fisher. The semi-structured interview schedule can be found in the S1 Appendix.

Target species were identified using photo based fish identification guides generated prior to interviews based on a list of species found in Bali [52]. A list of the known mangrove-associated fish were selected and compiled from this source, along with photographs from FishBase [37]. Fishing locations were recorded through participatory mapping during interviews. A paper map of the Perancak Estuary and the surrounding waters were annotated by fishers, also specifying differences in targets, fishing methods or seasonality of fishing sites as well as their location in relation to the mangrove. Fishing locations drawn on paper maps were digitised using ArcGIS.

Interview transcripts were analysed through categorization within Atlas.ti qualitative coding software, using an interpretive indexing approach [53]. This approach first outlines descriptive codes, which were based on the overarching research questions based on the semi-structured interview (S1 Appendix). At a second stage, analytic codes were derived, based on themes identified throughout the process. Following the decision that descriptions of the mangrove-fishing within the region could be best separated by fishing sector, descriptors of mangrove-fishing were drawn out for each sector respectively across 4 themes.

## 3 Results

**Fig 2:**
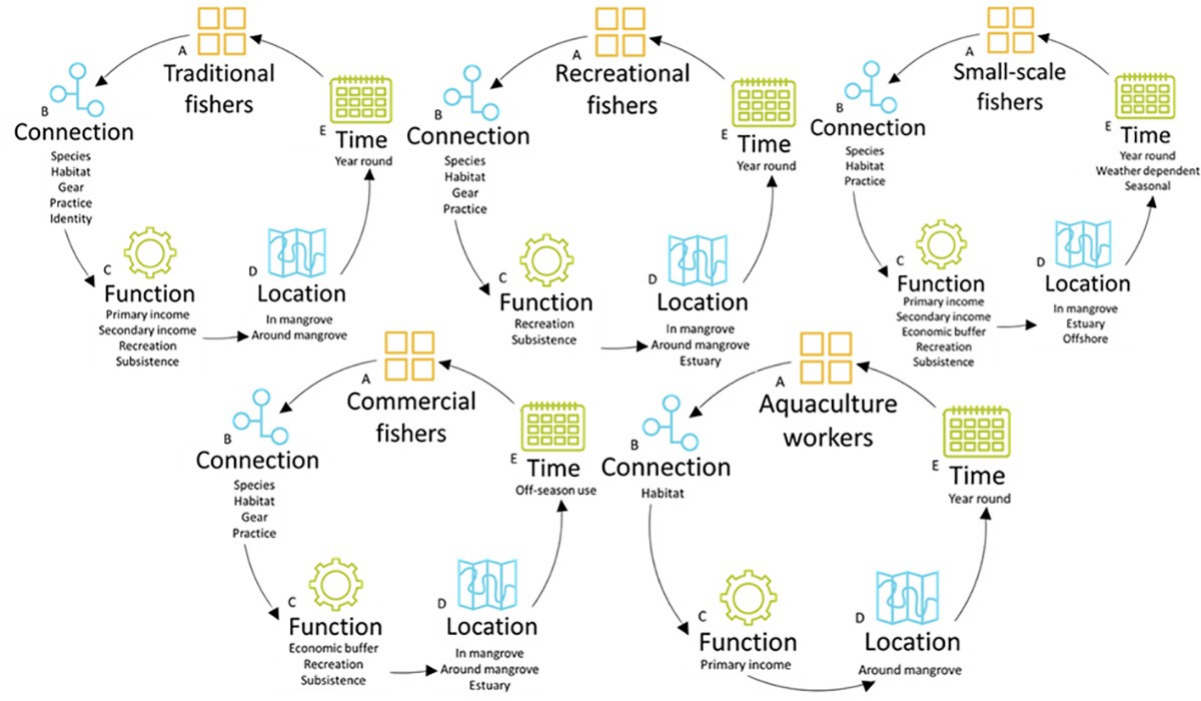
The typology of the Perancak Estuary mangrove-fishery, describing how fishers use mangroves, by A) sector, through dimensions of B) connection with the mangrove, C) function of fishing and D) location of fishing and E) time scale of mangrove-associated fishing.

In the Jembrana sub-district there are 5 sectors that are connected with mangroves for fish production. These are: traditional fishers; recreational fishers; small-scale fishers; commercial fishers and aquaculture workers. Four themes distinguished the characteristics of mangrove-fishing for each of these sectors: i) their connection to the mangrove; ii) the location of fishing; iii) the time of mangrove use; and iv) the function of mangrove-fishing for the individual. Fig 2 represents a typology of mangrove-fishing in the Jembrana sub-district, by sector, for each of these themes.

### Traditional fishers

#### Connection

People who go to the mangrove to fish or gather use a range of artisanal techniques and are referred to here as traditional fishers. These are the only group who identify as mangrove-fishers or “people who go to the mangrove”. There is however no specific local phrase to describe mangrove-fishers as the practiced activities are highly diverse. All species of fish and invertebrates targeted by traditional fishers are known mangrove-associates or are caught in the mangrove (Table 2). Fishing gears are individually specified towards catching a particular species in the mangrove, fulfilling each traditional fisher’s niche within the community (Table 2).

**Table 2:** Species caught by fishers of all sectors in the Perancak Estuary region of the Jembrana sub-district, Bali, their Mangrove Association (MA), yes (Y) or no (N), along with the fishing sectors of respondents who caught them (by self-identified sector) and the locations in which they have been caught across all sectors.

Group	Species name	Common name	MA?	Primary sector of respondents	Locations caught	N
Fish	*Lutjanus argentimaculatus*	Mangrove red snapper	Y	Artisanal Recreational Small-scale	Mangrove Estuary Offshore	9
Fish	*Rasterlliger brachysoma*	Short bodied mackerel	N	Artisanal Recreational Small-scale	Mangrove Estuary Offshore	6
Fish	*Lates calcarifer*	Barramundi	Y	Artisanal Recreational Small-scale	Mangrove Estuary	6
Fish	*Caranxsex fasciatus*	Big-eye trevally	Y	Artisanal Recreational	Mangrove Estuary	4
Fish	*Mugil cepahalus*	Mangrove mullet	Y	Artisanal	Mangrove	4
Fish		Grouper (general)	Y	Artisanal Recreational Small-scale	Mangrove Estuary Offshore	5
Fish	*Oreochromis mossambicus*	Mozambique tilapia	Y	Artisanal	Mangrove	3
Fish	*Oreochromis niloticus*	Nile tilapia	Y	Artisanal	Mangrove	1
Fish	*Leithognathus equulus*	Common ponyfish	Y	Artisanal Recreational	Mangrove	2
Fish	*Lutjanus grisius*	Grey snapper	Y	Artisanal	Mangrove	1
Fish	*Lutjanusrussellii*	Russel’s Snapper	Y	Artisanal Recreational	Mangrove	2
Fish		Snapper (general)		Recreational	Mangrove Estuary	3
Fish	*Saurida nebulosa*	Clouded lizardfish	Y	Artisanal	Mangrove	1
Fish	*Chanos chanos*	Milkfish	Y	Artisanal Small-scale	Mangrove Offshore	2
Fish	*Siganus lineatus*	Goldenlined spinefoot	Y	Artisanal	Mangrove	1
Fish	*Siganus guttatus*	Orange-spotted	Y	Artisanal	Mangrove	1
Fish	*Siganus vermiculatus*	Vermiculated spinefoot	Y	Artisanal	Mangrove	1
Fish	*Upeneus tragula*	Freckled goatfish	Y	Artisanal Recreational	Mangrove	2
Fish	*Terapon jarbua*	Jarbuaterapon	Y	Recreational	Mangrove	1
Fish	*Lethrinus atkinsoni*	Pacific yellowtail emperor	Y	Recreational	Mangrove	1
Fish	*Epinephelu srivulatus*	Halfmoon grouper	Y	Recreational	Mangrove	1
Fish		Grey shark (no species given)		Recreational	Estuary	1
Fish	*Auxis rochei*	Bullet tuna	N	Small-scale Commercial	Offshore	6
Fish	*Leptura canthussavala*	Savalai hartail	N	Small-scale	Offshore	4
Fish	*Sardinella lemuru*	Bali Sardinella	N	Small-scale Commercial	Offshore	3
Fish	*Decapterus macrosoma*	Short-fin scad	N	Small-scale, Commercial	Offshore	3
Crustaceans	*Scylla serrata*	Mangrove crab	Y	Artisanal Recreational	Mangrove	6
Crustaceans		Crab (general)		Recreational Small-scale Commercial	Mangrove Estuary	3
Shrimp		Shrimp (general)		Artisanal	Mangrove	2
Gastropods		Snails (general)		Artisanal	Mangrove	1
Bivalves		Shells (general)		Recreational	Mangrove	2
Bivalves		Scallops (general)		Small-scale	Mangrove	1
Bivalves		Oysters (general)		Artisanal	Mangrove	1
Bivalves	*Pernaviridis*	Green mussels	Y	Artisanal Commercial	Mangrove	3
Bivalves	*Polymedosa expansa*	Broad geloina/Marsh clam	Y	Artisanal	Mangrove	1
Cephalopods	*Loligo vulgaris*	Common squid	N	Small-scale	Offshore	1

N = number of respondents who reported catching the species.

#### Location

**Fig 3:**
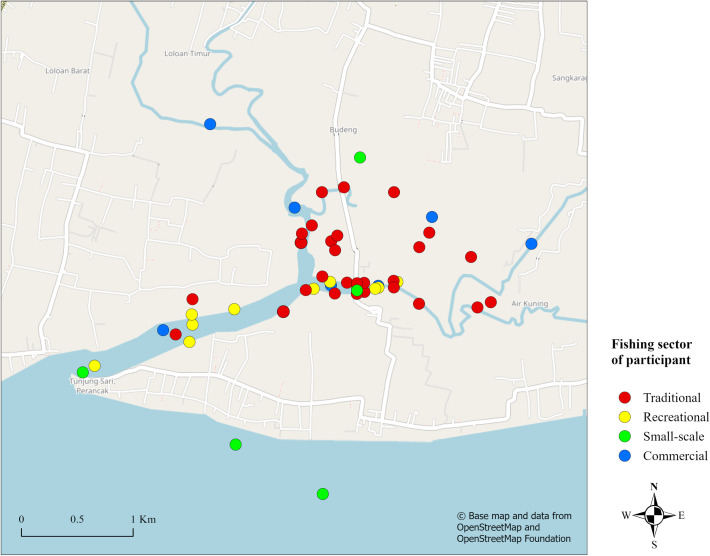
Fishing location by sector based on participatory mapping by fishers and other stakeholders in the Perancak estuary area of the Jembrana Sub-district, Jembrana Regency, Bali. Sectors included are traditional, small-scale, commercial and recreational fishing. For the location of the Perancak estuary in Bali see Fig 1. Sectors refer to groups of fishers who identify as belonging to the sector, as opposed to representing activity of the sector itself. The base map and data were sourced from OpenStreetMap and OpenStreetMap Foundation. This figure contains information from OpenStreetMap and OpenStreetMap Foundation, which is made available under the Open Database License. This map was created using ArcGIS Pro software.

Fishing takes place directly within the mangrove forest or on its muddy banks at low tide (e.g. gathering for mangrove crabs or mussels), or in the estuary within 1–6 m distance of the mangrove (e.g. fish or shrimp) (Fig 3). Inactive aquaculture ponds are sometimes used as secondary fishing sites when fishing in mangroves is unsuccessful. The location of traditional fishing grounds is not influenced by seasonality.

#### Time

*Traditional fishing is year-round*. *Fishing effort ranges from 3–9 hours per day when fishing is not limited by* the weather. Under normal weather conditions fishers will visit the mangrove daily but in the bad season fishing is typically reduced to two days per week.

#### Function

Traditional fishing offers a low income, but year-round, occupation. It comprises the sole occupation or the primary occupation where fishers hold secondary seasonal or part time occupations. These seasonal occupations are generally secondary to fishing in terms of household income although in some cases, for example work in construction or aquaculture, it can form the dominant proportion of monetary income. Most fishers keep a little of the lower quality catch for subsistence; some fishers can maintain all of their household seafood needs from fishing. Some traditional fishers also partake in additional recreational fishing on weekends or evenings with their family. This additional catch is kept only for subsistence.

### Recreational fishers

#### Connection

Recreational fishing is commonly conducted as a “hobby” by those who are either retired or currently work in non-fishing occupations. As such, recreational fishers do not self-identify as mangrove-fishers, or as fishers at all, as fishing is not their occupation. However, all species caught by recreational fishers are known mangrove associates (Table 2). Snappers (*Lutjanus* spp.), and particularly mangrove red snapper (*Lutjanus argentimaculatus*), are the most sought after target catch amongst recreational fishers. Some bait collection for recreational fishing involves the practice of going to the mangrove for gathering. However most recreational activity uses hand lines and targets demersal fish found in the Perancak Estuary or smaller tributary rivers.

#### Location

Recreational fishing is concentrated on one bridge crossing the Perancak estuary (Fig 2). When fishing from the bridge, fishers are no more than 30 metres away from the mangrove. Some fishers also stand in the river or on the river bank, between 2 and 12 metres from the mangrove but rarely within the mangrove forest itself. Fishers choose their distance from the mangrove for logistical reasons related to gear configuration, for example so that fishing lines do not become entangled within mangrove roots.

#### Time

Recreational fishing occurs year round, only being influenced by changeability in the weather. Time spent fishing ranges between 1–5 hours per trip and between 2–7 trips per week.

#### Function

Catch is kept only for personal consumption and thus has subsistence value. For some households, the fish caught during recreational fishing fulfils all of their intake of fish in the diet.

### Small-scale fishers

#### Connection

Mangrove-associated species are not the primary target species for small-scale fishers. However, the availability of their primary target species, Bali sardinella (*Sardinella lemuru*), fluctuates seasonally and therefore small-scale fishers intermittently target demersal fish offshore which include mangrove-associated species (Table 2). Further, when the weather prevents offshore fishing activities, small-scale fishers also fish within the mangrove-estuary. During this time they catch mangrove-associated fish species (Table 2); how often this option is used was not clear from this study. Small-scale fishers do not practice mangrove gathering or use gear specific to fishing in the mangrove during this time.

#### Location

**Fig 4:**
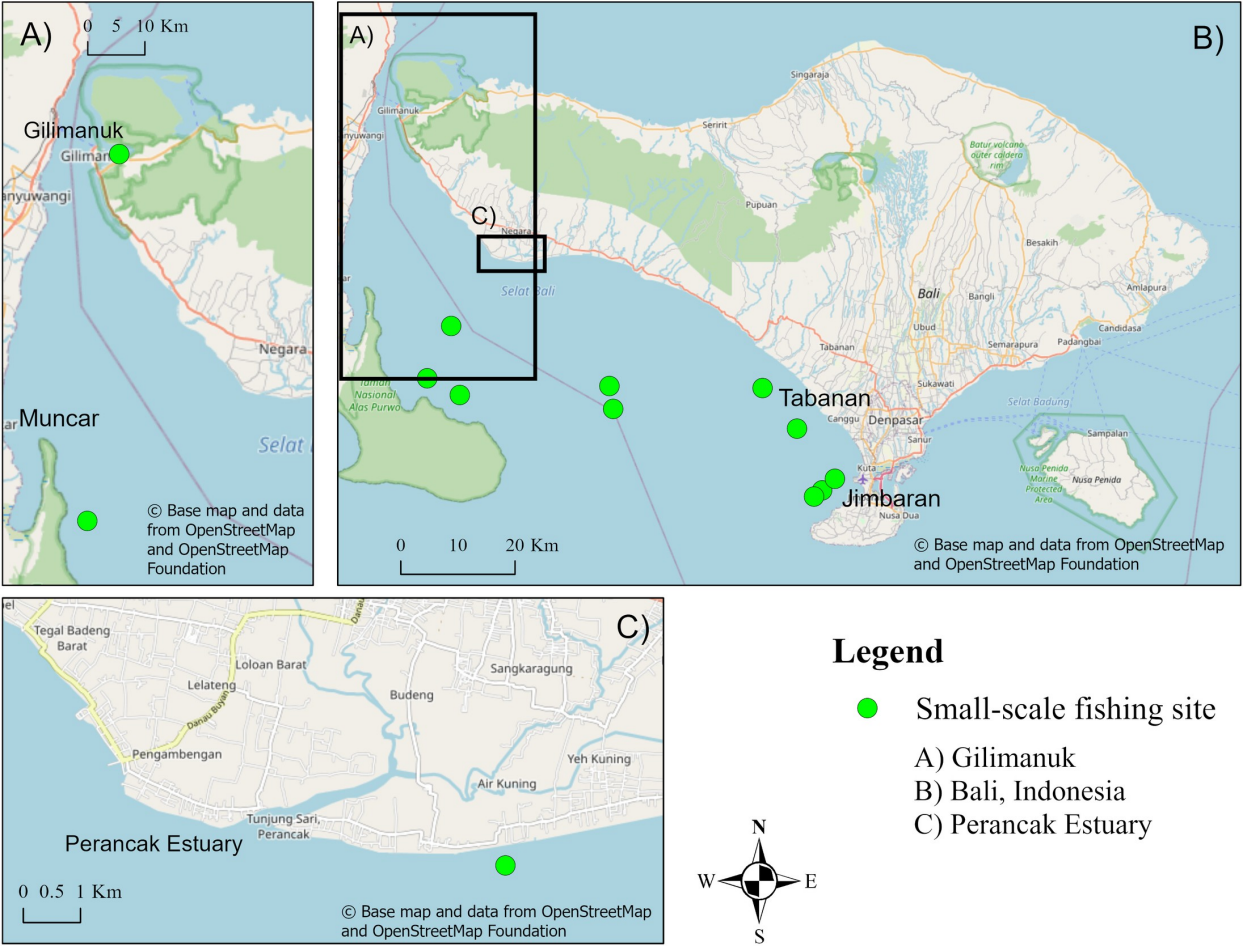
Offshore fishing sites as annotated by small-scale fishers in the Jembrana sub-district, Jembana Regency, Bali. Fishing sites are marked as the mid-point of polygons and lines drawn by fishermen. Map showing A) Gilimanuk, West Bali, B) Bali, Indonesia and C) The Perancak Estuary, Jembrana, West Bali which was the primary study site. The base map and data were sourced from OpenStreetMap and OpenStreetMap Foundation. This figure contains information from OpenStreetMap and OpenStreetMap Foundation, which is made available under the Open Database License. This map was created using ArcGIS Pro software.

According to regulation, small-scale fishing can take place within two zones which are bound at a minimum of 4 nautical miles (7.4 km) and 8 nautical miles (14.8 km) respectively from the Balinese coast [48]. Fishers had difficulty specifying their distance from shore when fishing, but it is noted that when fishing for demersal fish, they are closer to shore than when targeting pelagic fish. The locations that small-scale fishers frequent offshore, and thus catch some mangrove-associated fish (Table 2), are shown in Fig 4. The locations in the Perancak estuary used by small-scale fishers when weather prevents usual fishing are shown in Fig 3.

#### Time

Bali sardinella and other pelagics (non-mangrove associated) are targeted between May-December, while secondary demersal catches (mangrove associated) can be caught year-round. Switching between these catches means small-scale fishing can be a year-round occupation.

#### Function

Mangrove-associated catches contribute to both primary and secondary income for small-scale fishers, as well as providing an economic buffer when weather prevents usual fishing activities. Fishing also performs a secondary subsistence purpose as fishers keep some catch for consumption. Small-scale fishers also occasionally use the mangrove area for recreational fishing activities, keeping the catch for consumption.

### Commercial fishers

#### Connection

Commercial fishing activity does not target mangrove-associated species (Table 2). It is always conducted offshore and never uses the mangrove habitat. However, commercial fishing activities are seasonal and those fishers on commercial boats require secondary options during the off-season. During this time, fishing in the Perancak estuary can provide a secondary option for both subsistence and income, as discussed below. When using this option, commercial fishers adopt traditional practices of fishing in the mangrove and target mangrove-associated species, such as small fish and bivalves (Table 2). Some commercial fishers are therefore connected to the mangrove through the practice of going to the mangrove and using typically mangrove-associated fishing techniques. Notwithstanding this behaviour, commercial fishers did not identify themselves as mangrove fishers or traditional fishers.

#### Location

The fishing areas used for traditional fishing by fishers from the commercial sector in the bad season are displayed in Fig 2.

#### Time

Mangrove-associated fishing takes place during the bad season (in terms of catches) for commercial fishing, between May and October. However, at the time of the surveys (2017), there had been no commercial fishing for 9 to 12 months due to an adverse fluctuation in Bali sardinella stocks. As the bad season can span from 6 months to an entire year, it represents a wide time period through which fishers may be dependent on this secondary option for subsistence or income from fishing

#### Function

During the bad season for commercial catches, fishers obtain products from the mangrove estuary either for subsistence or for sale, as an economic buffer to their commercial fishing income.

### Aquaculture workers

#### Connection

Aquaculture production includes white leg shrimp (*Litopenaeus vannamei*) known locally as “Vannamei”, black tiger shrimp (*Penaeus monodon*), known as “Windu” or “Butang”, and milkfish (*Chanos chanos*). This production derives no direct benefit through mangrove-fishery enhancement. However, it is included here as aquaculture workers unanimously suggested that the presence of mangrove habitat was indirectly linked to successful production of fish and shrimp within aquaculture ponds.

Aquaculture in the sub-district uses mostly traditional technology, although wealthier pond owners use manual (semi-intensive) technology. The use of aerators (to enrich the pond water with oxygen) distinguishes semi-intensive from traditional ponds. Both methods rely on inputs of water from the Perancak Estuary or its tributaries. The value of the presence of mangrove is thought to be through the filtering properties of the trees, preventing polluted river water from entering the ponds. Disease sharing between ponds is thought to be the largest risk to production in the sub-district, with farms all using the same water channels as inlets and outlets for their ponds. Aquaculture workers therefore actively plant mangrove, or rehabilitate mangrove habitat, on the periphery of their ponds. The connection that aquaculture workers or owners have with the mangrove is with use of the habitat only.

#### Time

Aquaculture production is active year-round. However, there have been notable variations in production from year to year. Events in 2005 and 2010 were recounted by respondents as dramatically reducing, or even completely halting, production. The 2005 hiatus was attributed to river water of poor quality entering the ponds, caused by dredging of sediments, subsequent poor river quality and degradation of mangroves, and therefore reduction of the filtering function. These impacts were perceived as being exacerbated by mangrove cutting. Respondents suggested that 70% of aquaculture in the region was inactive during this time and was halted for several years (until 2014 in some cases) or resulted in a switch to other more disease resistant culture species such as milkfish, or from black tiger shrimp to white leg shrimp, now the most popular shrimp species farmed in the area.

In the 2010 halt to production, respondents suggested that reduction of river quality was caused by potassium fishing in the rivers, prompted by poor catches offshore. Respondents also attributed changes in production to agricultural run-off, sharing of disease between ponds, changing regulations regarding culture species and use of antibiotics. It should therefore be noted that changes to mangrove area are not the only influence on aquaculture production. Nonetheless, of all the sectors interviewed, respondents from the aquaculture sector appeared to hold the strongest perception of the importance of mangrove presence to their own financial wellbeing.

#### Location

Ponds line the estuary and the surrounding rivers and their tributaries. Those ponds that use water inputs from these waterways are bordered by mangroves. Ponds also have planted mangroves around the barriers of ponds for structural support of the earthen banks.

#### Function

Aquaculture work is the primary source of income for pond owners or workers. Many of the aquaculture ponds are family-run businesses.

### 3.1 Wider Bali

**Fig 5:**
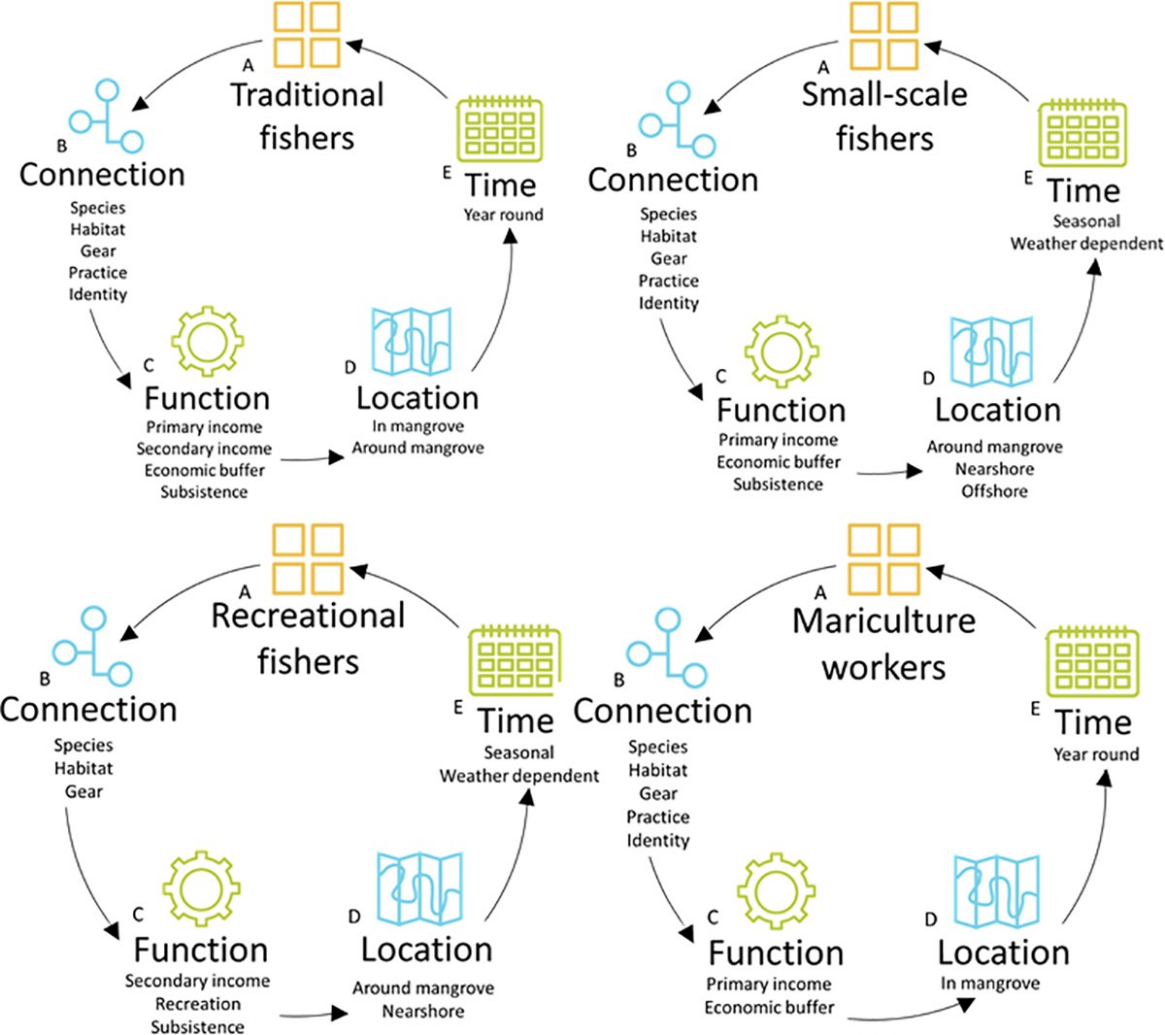
A typology of mangrove-fishing by the Benoa Fishing Community, by A) sector, through B) connection with the mangrove, C) function of fishing and D) location of fishing and E) time of mangrove-associated of fishing.

**Fig 6:**
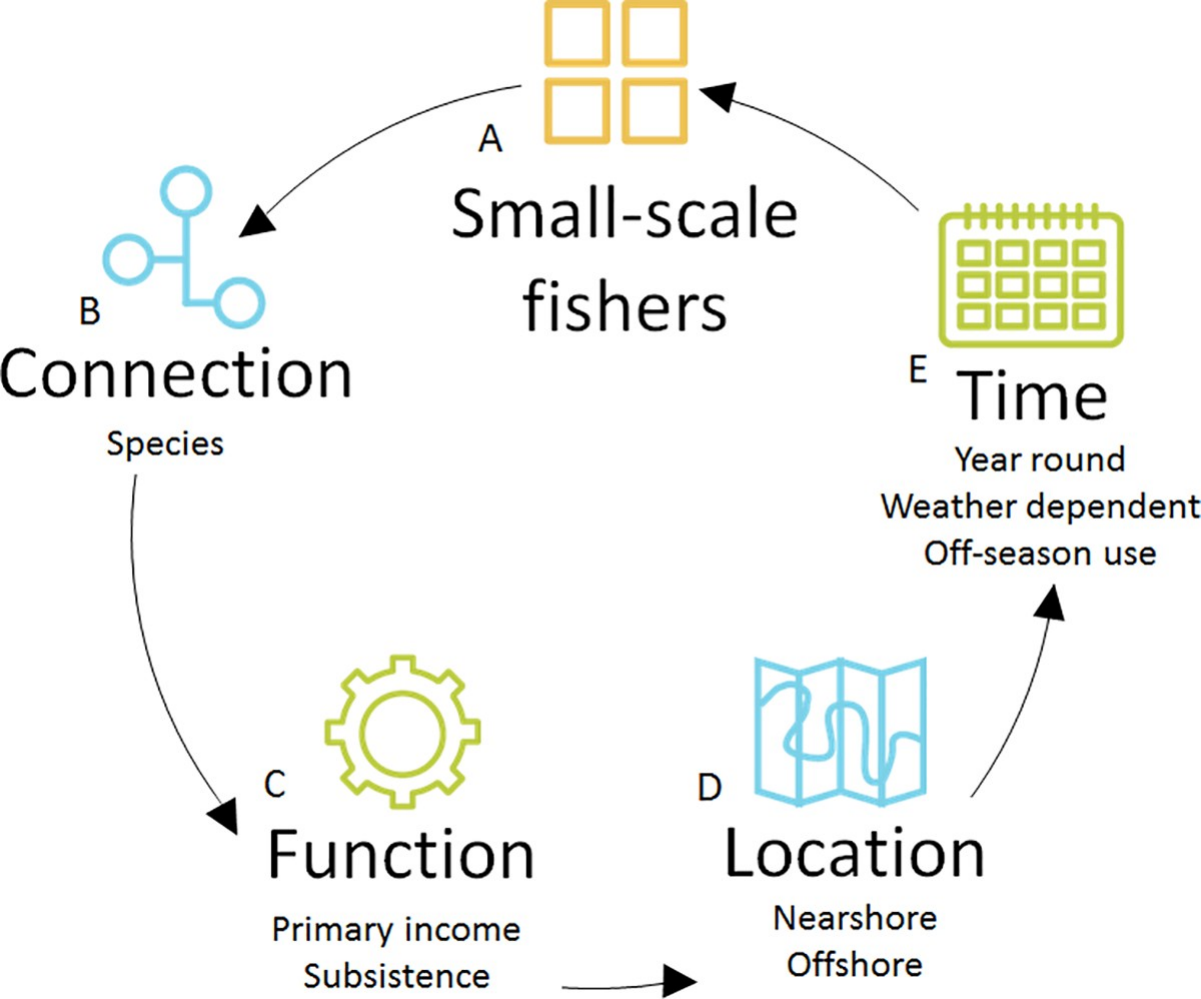
A typology of mangrove-fishing by the Gilimanuk Fishing Community by A) sector (small scale fishers only) through B) connection with the mangrove, C) function of fishing and D) location of fishing and E) time of mangrove-associated of fishing.

Fishers from 4 sectors are connected to the mangrove in Benoa. These are traditional fishers, small-scale fishers, recreational fishers and mariculture workers. Mariculture (aquaculture which takes place within the marine environment) in Benoa involves the culture of mangrove crab (bought externally) in enclosed sections of the mangrove forest. In Gilimanuk, small-scale fishers only have a connection to the mangrove. Figs 5 and 6 show the typology of the mangrove-fishing in the Benoa and Gilimanuk fishing communities respectively.

### 3.2 A framework for characterizing mangrove-fisheries

Fig 7.

**Fig 7:**
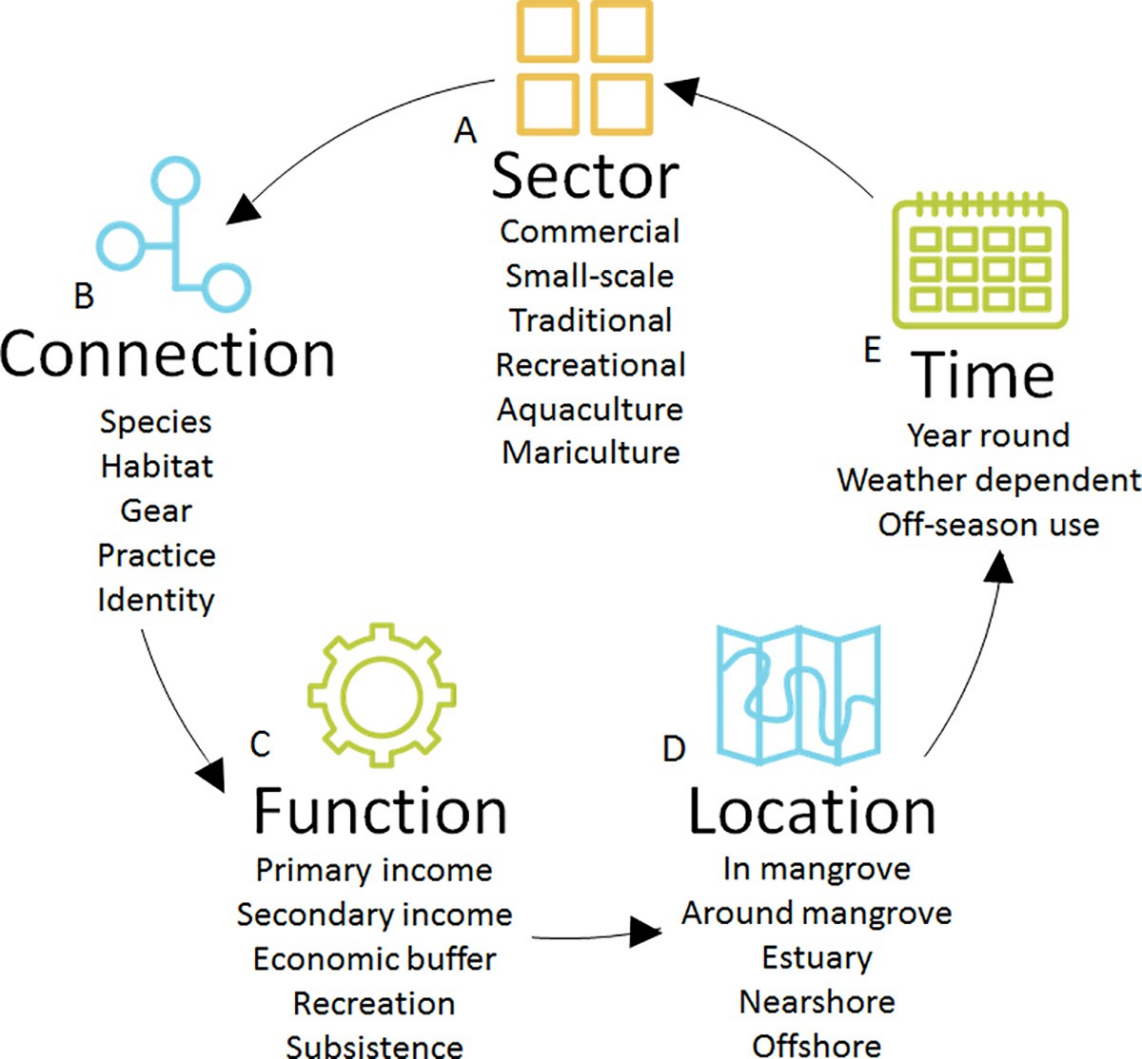
A framework for characterising mangrove-fishing in a local context. Following the diagram anti-clockwise, the typology of a mangrove-fishery can be drawn out, by A) sector, through themes of B) connection to the mangrove by fishers of that sector, C) the function that mangrove-associated fishing has for those individuals, D) the location in which mangrove-associated fishing takes place and E) the time at which those fishers use or benefit from the mangrove.

## 4 Discussion

### 4.1 Defining the Perancak Estuary Mangrove-Fishery

Identifying the complexity and fluidity of the 5 sectors that carry out fishing or cultural activities in the Perancak Estuary shows that each group in the region, even those with less obvious connections with the mangrove, derive some benefit from the mangrove in some or all of their activities. The mangrove-fishery in Bali is multi-dimensional in terms of how fishers are connected to the mangrove, the function that fishing has, the time during which fishers are conducting mangrove associated fishing and the locations of fishing. The connection individuals within any particular sector have with the mangrove is also variable and complex. The complexity of these sectors, their interaction with the mangrove and with each other can be linked to form a framework (Fig 7) which represents how mangrove-fisheries can be defined.

Interviewing fishers in Bali uncovered groups of fishers that are not recognised in the available official regional fishing reports. This reinforces studies elsewhere which document how small-scale fisheries or groups of fishers can be “invisible” to managers when such groups co-exist with larger scale or industrial fisheries [4, 6, 54]. Quite apart from providing a snap-shot view, interview surveys in this study also allowed for insights into changing environmental and socio-economic conditions and how these conditions relate to seasonal changes in fishing patterns. This allowed the framework developed to reflect the dynamic nature of mangrove-fisheries. For instance, fishers that use the mangrove only during bad seasons or when the weather is unsuitable for their primary mode of fishing allows fishers security in earnings and livelihoods.

Diversity in mangrove use can provide an important economic buffer to large-scale offshore fishing activities. That commercial fishers from the region endured a 9-month period of no fishing in 2016/17 suggests that the offshore fishery is not in a stable condition. Furthermore, the number of active offshore fishing boats far exceeds the maximum number of vessels permitted by Regency management targets. Under the Government of East Java and Bali, just 83 Balinese vessels operating purse seines are permitted. However, in 2014, 2,464 active vessels (with outboard motors) were counted [48]. Current national management does not address the issue that continued periods of low production in the commercial fishery could result in fishers more regularly turning to mangrove estuary resources as an alternative occupation. Consequently, the importance of mangrove-fishing for the small-scale and commercial fishing sectors could increase with increasing pressure on offshore stocks. This is important to monitor, as increased resource use within the Perancak Estuary could have implications for other fishers, such as traditional and recreational fishers, who are already reliant on resources there.

No comprehensive landings data, other than for the commercial fishing sector, exists for the Jembrana sub-district. Therefore, it was not possible to quantify the contribution of mangrove-associated catch to the livelihoods of each of the stakeholders identified or gauge the total number of stakeholders. The aim of this paper, however, was to identify the gap in knowledge as to what mangrove-fisheries can encompass, rather than to expose the quantitative value of further hidden catch. The result has been to enhance our understanding of the socio-ecological link between mangroves and fishers, to be further built upon through wider geographical studies of mangrove-fishing communities.

### 4.2 How does the Perancak Estuary Mangrove-Fishery compare to others in Bali and elsewhere?

Three different typologies describe mangrove use by fishers in the Jembrana sub-district (Perancak Estuary), Benoa and Gilimanuk (Figs 2, 5 and 6). These communities are located just 20–80 km apart and therefore it can be argued that a single definition of what mangrove-fisheries encompass cannot, and should not, be applied from one location to another, even if they are in close proximity. There were similarities between communities, with traditional and small-scale sectors in Jembrana and Benoa carrying out similar activities pertaining to similar livelihood functions. However, interviews in Benoa uncovered an additional sector, mariculture, which uses mangroves, which was not observed in Jembrana. Further, interviews in Benoa suggested that recreational fishing, when conducted for tourism, can be used as an income source, whereas it was used only for recreation and for subsistence in the Jembrana community. It is therefore important to look at the function that mangrove-fishing has within sectors, as well as the presence of the sectors themselves, to understand the societal importance of various fishing activities.

Connection to the mangrove within the Gilimanuk fishing community appeared less multi-dimensional than that of fishers in Jembrana and Benoa, being limited to catching mangrove-associated fish species offshore and around other coastal habitats (Fig 6). The activities of the Gilimanuk fishing community in the past, as described in 1983 [52], were much more diverse. Changes are likely to have been influenced by extension and zonation of the Bali Barat marine reserve in the area, focussed on reducing damage to mangroves as well as ceasing destructive fishing methods [52]. In comparison, no specific management regarding mangrove-use for fishing (other than direct cutting of mangroves) appeared to exist in Jembrana or Benoa. Levels of governance of a mangrove area might therefore influence what mangrove-fisheries can encompass.

Comparing the Perancak Estuary Mangrove Fishery to mangrove-fisheries as they have been described in previous evaluations (Table 1), this mangrove-fishery appears more complex. An example of this is the diversity of groups involved in mangrove-fishing, which in the Perancak Estuary Mangrove-Fishery involves actors from 5 different sectors, while those which have quantified mangrove-fishery value previously have involved just 2 sectors at most (Table 1). The complexity of functions that mangrove-fishing has for fishing livelihoods in the Perancak Estuary was also greater compared to the prior literature which has generally stated only incomes or biomass generated through mangrove-fishing, not distinguishing its contribution to secondary or economic buffer incomes, subsistence or recreation. Moreover, the temporal and spatial variability in mangrove-fishing displayed in the typology developed has not been conveyed elsewhere in the literature regarding other mangrove-associated fisheries. As such, mangrove-fisheries as they have been described thus far do not capture the full range of what mangrove-fisheries can encompass, as shown by the typology developed in this study.

This initial comparison explores the representation of mangrove-fisheries from quantitative studies of mangrove-fishery value (Table 1). Examples from socio-ecologically focussed or qualitative studies of mangrove-fisheries, however, describe mangrove-fisheries with complexity much more comparable to our findings in Bali. For instance, in the Ciénaga Grande de Santa Marta lagoon system in the Colombian Caribbean, mangrove-fishing has been observed to vary spatio-temporally and to involve a number of fishing gears and targets [55]. Studies from the qualitative literature on mangrove-fisheries have also described additional sectors that benefit from mangroves that were not observed in Bali, for example those involved in fishery processing and trading in the Sundarbans, Bangladesh [34]. In the Caeté Estuary, Brazil, an additional function that we did not observe, using mangrove-fishery for emergency food provision, was reported. Whilst complex, the Perancak Estuary Mangrove-Fishery cannot represent all of the possible characteristics and interactions that mangrove-fisheries can exhibit, as identified in descriptions of other mangrove-fisheries in Bali and elsewhere. The mangrove-fishery studied in the Perancak Estuary, therefore, might not demonstrate the upper end of complexity of all mangrove-fisheries. Further research should therefore apply this framework in other regions to develop a wider geographic understanding of mangrove-fishery interactions.

## 5 Conclusions

Justifications for mangrove-fishery management, or simply mangrove conservation, have been attempted through many mangrove-valuation studies [30, 50, 51]. However, these studies often focus upon a single dimension of mangrove use by a community. Under-valuation of mangroves is considered one of the leading causes of mangrove conversion to other land uses [52]. This case study of a relatively small mangrove-fishery suggests that a mangrove-fishery can encompass more complex interactions, and therefore display greater societal importance, than represented in many measures of mangrove-fishery value. This study should therefore encourage the holistic characterisation of mangrove-fisheries in other countries, prior to trade-off decisions over mangrove management or land use. Moreover, it should encourage researchers and managers to look outside of the groups of fishers traditionally expected to benefit from mangrove-fishing, leading to a broader definition of mangrove-fisheries in each local context. This is particularly pertinent where offshore fisheries are declining and small-scale fisheries may be offering an economic and ecological buffer which is likely to be underestimated, or even invisible, within fisheries or mangrove management strategies.

It has been stressed that knowledge of the actual uses of mangroves in a community, rather than the assumed uses of mangroves, are essential to sustainable community-based management [56]. The framework in this study, therefore, presents a first step in the direction of mangrove-fishery management in which mangrove-fisheries are holistically characterized, prior to next step of evaluating societal or monetary value. Thus, all stakeholders, fishing activities and vital ecosystem services that those groups rely upon are recognized in management decisions. This will lead to more informed trade-off decisions over resource use and ultimately help towards meeting ecological and societal sustainability targets.

## Supporting information

S1 Appendix Semi-structured interview transcript used to interview fishers about mangrove use in Bali. (DOCX)
