# Supplementary material for: Defining mangrove-fisheries: A typology from the Perancak Estuary, Bali, Indonesia
Source: PLoS One. 2021 Apr 21;16(4):e0249173. doi: 10.1371/journal.pone.0249173 (PMC8059815; doi:10.1371/journal.pone.0249173)
Supplement: S1 Appendix — (DOCX) [file pone.0249173.s001.docx]

**S1 Appendix. Semi-structured interview transcript used to interview fishers about mangrove use in Bali.**

| Respondent has confirmed that they give consent to be interviewed and for the discussion to be recorded and used in the research project described? |  |
| --- | --- |
| Fishing sector |  |
| Age |  |
| Gender |  |
| Family size |  |
| How long have you been fishing? |  |
| How did you start fishing? Who taught you to fish? |  |
| Do you always fish alone/ who do you fish with? |  |
| Are you family involved in fishing? |  |
| How much time do you spend fishing? In the  good season and the bad season? |  |
| What is your household income from fishing? How much do you make from fishing? |  |
| Do you go the market to sell the fish or do you keep most for yourself and your family to eat? |  |
| How much of the fish you catch do you sell? |  |
| How much of the fish you catch do you eat? |  |
| Is your catch similar to the other fishers? What are the most profitable catches? |  |
| Do you think a lot of your diet comes from fish/seafood in Bali? Do you also go out to eat out or buy other foods from the market? |  |
| What kind of fishing gear do you use? | Other: |
| Hook and line Gill net Spear fishing Payang (pelagic Danish net) Bagan (lift net)  Troll line Hand line Purse seine Cast net Beach seine Tuba  Gathering by hand | |
| Where do you fish? And why do you fish there? (Economic, ecological or social reasons?) |  |
| Which fish do you usually catch?  How much? |  |
| Lemuru (Bali sardinella)  Cakalang (Skipjack tuna)  Layang (Scad)  Belanak (Mangrove mullets)  Tongkol (Frigate tuna)  Selar (Trevallies)  Cumi-cumi (Common squids)  Tembang (Fringescale)  Layur (Hairtails)  Tenggiri (Narrow-barred spanish mackerel)  Kerapu (Grouper)  Kakap (Snappers)  Kembung (Short-body mackerel)  Tuna (Tuna) |  |
